# Boron homeostasis affects Longan yield: a study of NIP and BOR boron transporter of two cultivars

**DOI:** 10.1186/s12870-023-04689-8

**Published:** 2024-01-02

**Authors:** Jing Wang, Junbin Wei, Dongliang Guo, Xinmin Lv, Boyun Wang, Yiyun Wang, Jianguang Li

**Affiliations:** Key Laboratory of South Subtropical Fruit Biology and Genetic Resource Utilization, Ministry of Agriculture, Institute of Fruit Tree Research, Guangdong Academy of Agricultural Sciences, Guangzhou, 510640 China

**Keywords:** Borate, Boric acid, Exporter, Crossbreed, Translocation, Spatiotemporal expression

## Abstract

**Background:**

Essential micronutrient Boron (B) plays crucial roles in plant survival and reproduction but becomes toxic in higher quantities. Although plant cells have different B transport systems, B homeostasis is mainly maintained by two transporter protein families: B exporters (BOR) and nodulin-26-like intrinsic proteins (NIP). Their diversity and differential expression are responsible for varied B tolerance among plant varieties and species. Longan is a highly admired subtropical fruit with a rising market in China and beyond. In the present study, we cultured Shixia (SX) and Yiduo (YD), two differently characterized Longan cultivars, with foliar B spray. We analyzed their leaf physiology, fruit setting, B content, and boron transporter gene expression of various tissue samples. We also traced some of these genes' subcellular localization and overexpression effects.

**Results:**

YD and SX foliage share similar microstructures, except the mesophyll cell wall thickness is double in YD. The B spray differently influenced their cellular constituents and growth regulators. Gene expression analysis showed reduced BOR genes expression and NIP genes differential spatiotemporal expression. Using green fluorescent protein, two high-expressing NIPs, NIP1 and NIP19, were found to translocate in the transformed tobacco leaves' cell membrane. NIPs transformation of SX pollen was confirmed using magnetic beads and quantified using a fluorescence microscope and polymerase chain reaction. An increased seed-setting rate was observed when YD was pollinated using these pollens. Between the DlNIP1 and DlNIP19 transformed SX pollen, the former germinated better with increasing B concentrations and, compared to naturally pollinated plants, had a better seed-setting rate in YD♀ × SX♂.

**Conclusion:**

SX and YD Longan have different cell wall structures and react differently to foliar B spray, indicating distinct B tolerance and management. Two B transporter NIP genes were traced to localize in the plasma membrane. However, under high B concentrations, their differential expression resulted in differences in Jasmonic acid content, leading to differences in germination rate. Pollination of YD using these NIPs transformed SX pollen also showed NIP1 overexpression might overcome the unilateral cross incompatibility between YD♀ × SX♂ and can be used to increase Longan production.

**Supplementary Information:**

The online version contains supplementary material available at 10.1186/s12870-023-04689-8.

## Background

Boron (B) is one of the essential micronutrients that play multiple crucial physiological functions in higher plants, such as cell wall formation, cell division, cell elongation, flowering, fruiting, rooting, nutrient uptake, ion flux, carbohydrate metabolism, nitrogen metabolism, and hormone regulation [1]. However, high B levels are hazardous to plant development [2]. Different parts of a plant have unique requirements and functions of B [1]. Furthermore, various plant species, varieties, and plants of various ages respond differently to B shortage or toxicity [2]. The primary source of B in plants is from the soil, which can be found as borate or boric acid (B(OH)_3_) in soil solutions. Plants absorb boric acid using three different methods, depending on its accessibility: diffusion, major intrinsic proteins (MIP), and B exporters (BOR) transporters [3]. Passive diffusion and MIP transport are used when B is adequately available, whereas BOR transporters are used when B is absent. Previous studies revealed B transport in *Arabidopsis thaliana* plant occurs not just by passive diffusion but is also regulated by transport proteins such as boric acid channels (nodulin-26-like intrinsic proteins or NIPs) and B exporters (BORs) [4]. NIPs and BORs work together to keep plant development regular in high and low B environments.

Longan (*Dimocarpus longan* Lour.) is a prominent subtropical fruit tree native to South China, holding 70 and 60% of the world's Longan cultivation field and yield in 2010 [5]. There are over 400 varieties of Longan cultivars in China [6], and the majority (68%) of them grow during the mid-season (August-September) [5]. This season is presided by the rainy season of southeast Asia that depletes relatively soluble B in the soil by leaching it with the rainfall [2]. B effect on Longan's fruit quality is well studied and practised [5]. A foliar spray of boric acid and applying B fertilizer have increased fruit size, quality, and soluble sugar [3] and reduced flower and fruit drops [7]. For optimal Longan harvesting, it is necessary to understand and employ the proper dose of B. However, the molecular mechanism for B uptake and translocation in various Longan species are not fully understood. We studied two commonly cultivated Longan varieties, the traditional cultivar Shixia (SX), with 140 years of cultivation history in China [5], and the newer Yiduo (YD) cultivar, with better fruit quality [6, 7]. YD was introduced to China from Thailand, making up 90% of the Thai Longan yield [7].

In this study, we observed cellular structural differences, the sugar content of their leaf cells, the flower's Jasmonic acid (JA) content, the fruit-setting rate, and the B content of different plant organs after foliar B treatment. We also identified the genes of the BOR and NIP gene families and studied their evolutionary relation, cis-acting elements, and level of expression in different tissues. Depending on this analysis, genes that expressed most in various organs of SX and YD were selected for further study. Subcellular localization of this gene product was studied by creating versatile green fluorescent protein (GFP)-gene expression vectors and transforming SX pollen. B tolerance of the GFP-gene transformed pollens was studied, and Longan plants were pollinated using this pollen to check the germination rate. The unilateral cross incompatibility (UCI) between SX and YD was reported to hinder the attempt to generate successful crossbreeding [6]. So, we established a crossing of YD♀ × SX♂ using hybrid pollen to study the fruit-setting rate of the hybrid panicle.

## Result

### Leaf phenotyping, structural and ultrastructural differences

**Fig. 1:**
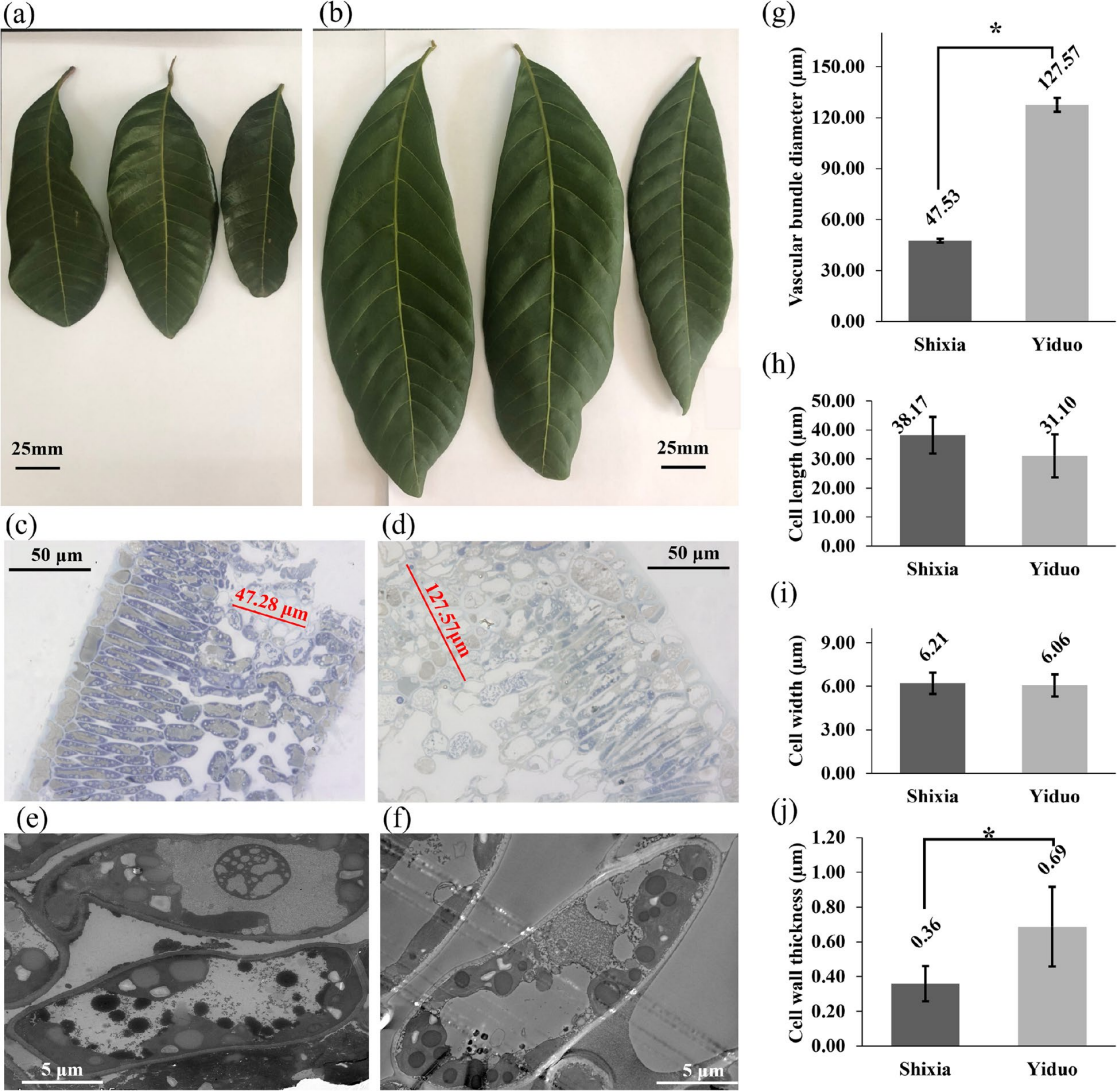
Leaf morphology of YD and SX Longan cultivars. Mature leaves of SX (**a**) are smaller than the YD (**b**) leaves. Under a light microscope, the semi-thin and longitudinal sections of SX (**c**) and YD (**d**) leaves show that the vascular bundle diameter is relatively more prominent in the YD leaves. Here, red straight lines represent vascular bundle diameters (**g**) measurements. TEM showed that SX (**e**) and YD (**f**) have similar cell lengths (**h**), and width (**i**), but the YD mesophile's cell wall is double in size from SX (**j**). The Y-axis in the graphs represents the measurements of the cell components in µm, the bars depict the mean ± standard deviation of SX and the YD biological replicates (*n* = 3), and the asterisks indicate significant differences between SX and YD samples at a significance of *p* ≤ 0.05 (*) in the student's *t-*test

Mature YD and SX, both cultivars of Longan, have oblong, blunt-tipped leaves with 6 to 9 pairs of leaflets per leaf in pinnately compounded and alternate formation. SX leaves were smaller (10–12 cm) (Fig. 1a) than the YD leaves (18–20 cm) (Fig. 1b). Under the light microscope, toluidine blue stained longitudinal sections of both leaves showed the diameter of the vascular bundles is more prominent in the YD leaf (Fig. 1d) than in the SX leaf (Fig. 1c, g). Transmission electron microscopy (TEM) of mesophyll cells of both foliage showed similar cell size (Fig. 1h, i), but the cell wall of the YD (Fig. 1f) is significantly thicker than the SX (Fig. 1e, j).

**Fig. 2:**
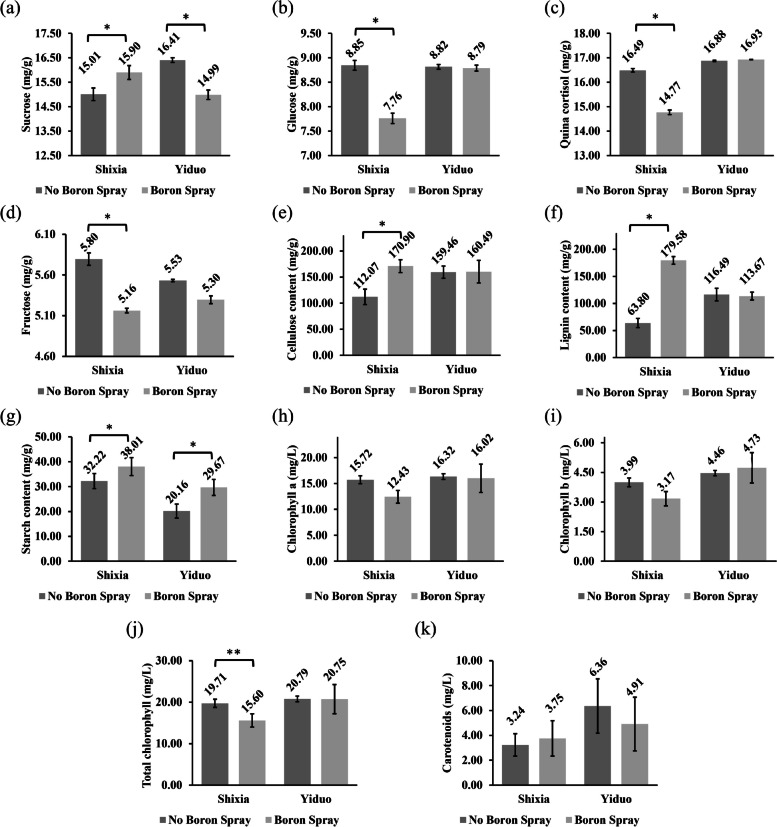
Effect of boron spray on the leaf constituents of YD and SX Longan cultivars. We measured the effect of foliar boron spray on leaf constituents such as **a** Sucrose, **b** Glucose, **c** Quina cortisol, **d** Fructose, **e** Cellulose, **f** Lignin, **g** Starch, **h** Chlorophyll a, **i** Chlorophyll b, **j** Total chlorophyll, and **k** Carotenoids. The Y-axis in these diagrams represents the concentration of respective leaf constituents, the bars depict the mean ± standard deviation of the biological replicates (*n* = 3) of SX and YD, with and without boron spray, and the asterisks indicate significant differences between boron treated and untreated SX or YD plants at a significance of *p* ≤ 0.05 (*), *p* ≤ 0.01 (**) in the student’s *t-*test

B influences various vascular plant functions, including root extension, indole-3-acetic acid oxidase, sugar translocation, carbohydrate metabolism, nucleic acid synthesis, and pollen tube formation, in different manners [8]. We also observed varied effects of the foliar B spray on some SX and YD leaf constituents. The sucrose content of the SX leaves significantly increased (15.01 mg/g to 15.90 mg/g). In contrast, the YD leaves substantially decreased (16.41 mg/g to 14.99 mg/g) (Fig. 2a). The concentration of glucose (8.85 mg/g to 7.76 mg/g) (Fig. 2b), Quina cortisol (16.49 mg/g to 14.77 mg/g) (Fig. 2c), and total chlorophyll (19.71 mg/L to 15.60 mg/L) (Fig. 2j) in SX leaves significantly decreased but didn’t change much in YD (8.82 mg/g to 8.79 mg/g, 16.88 mg/g to 16.93 mg/g, and 20.79 mg/L to 20.75 mg/L respectively). The fructose content decreased in both SX (5.80 mg/g to 5.16 mg/g) and YD (5.53 mg/g to g.30 mg/g) (Fig. 2d) but was more notable in SX. SX's cellulose (112.07 mg/g to 170.90 mg/g) (Fig. 2e) and lignin (63.80 mg/g to 179.58 mg/g) (Fig. 2f) content significantly increased, but the YD (159.46 mg/g to 160.49 mg/g, and 116.49 mg/g to 113.67 mg/g) remained relatively unchanged. The starch (Fig. 2g) content of both SX (32.22 mg/g to 38.01 mg/g) and YD (20.16 mg/g to 29.67 mg/g) significantly increased. The SX chlorophyll-a (Fig. 2h) and chlorophyll-b (Fig. 2i) content slightly decreased (15.72 mg/L to 12.43 mg/L and 3.99 mg/L to 3.17 mg/L, respectively) but was relatively unchanged in YD (16.32 mg/L to 16.02 mg/L and 4.46 mg/L to 4.73 mg/L respectively). The carotenoid (Fig. 2k) content increased in SX (3.24 mg/L to 3.75 mg/L) and decreased in YD (6.36 mg/L to 4.91 mg/L), but both were insignificant.

### Fruit-setting rate

**Fig. 3:**
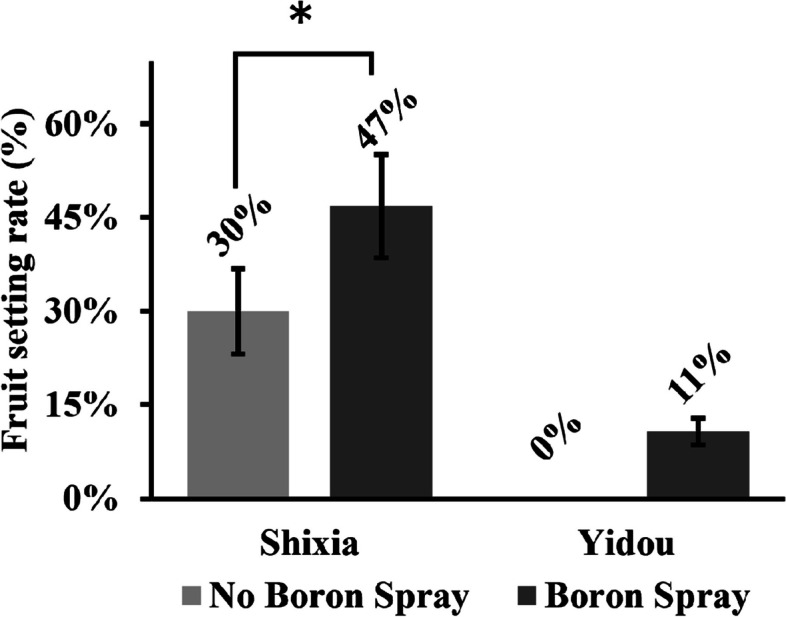
Foliar boron spray increases the fruit-setting rate in YD and SX Longan cultivars. The Y-axis of the chart represents the fruit setting rate in percentage, the columns show mean ± standard deviation of the biological replicates (*n* = 3) of both SX and YD, with and without boron spray, and the asterisks indicate significant differences between boron unsprayed and sprayed plant at a significance of *p* ≤ 0.05 (*) in student’s *t-*test

Fruit-setting was observed for SX and YD Longan varieties with and without foliar B spray (Fig. 3). Although the fruit-setting rate differed without B spray in SX and YD, the fruit-setting rate increased in both varieties after spraying but was significant in SX. The fruit-setting of B unsprayed and B sprayed SX were 30% and 47%, whereas in YD were 0% and 11%.

### Total boron content

**Fig. 4:**
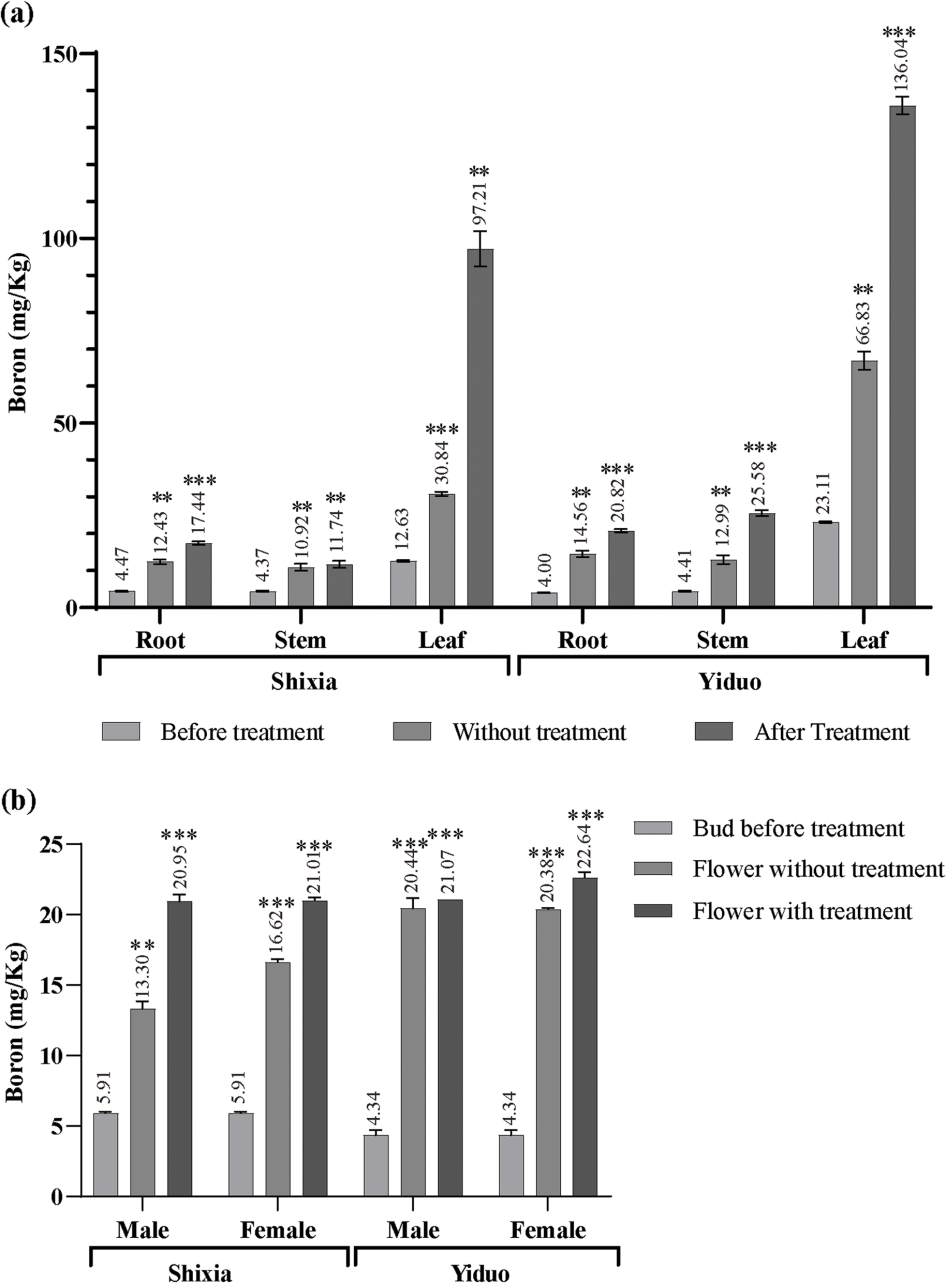
Effect of foliar B spray on the B content of Longan organs. The B content of SX and YD vegetative (**a**) and reproductive (**b**) organs increased after foliar B spray**.** The Y-axis of the chart represents the concentration of B, and the bars represent the mean ± standard deviation of biological replicates (*n* = 3) before B treatment, with B treatment and without B treatment of SX and YD Longan’s vegetative and reproductive organs (both male and female) and the asterisks indicate significant differences compared to before treatment replicates at *p* ≤ 0.005 (**), *p* ≤ 0.001 (***) in the 2-way ANOVA analysis

With foliar B spray, the B content of the root, stem, leaf (Fig. 4a), and flowers (Fig. 4b) significantly increases in both YD and SX Longan varieties. The root B content increased in SX from 12.43 mg/Kg to 17.44 mg/Kg and from 14.56 mg/Kg to 20.82 mg/Kg in YD (Fig. 4a). The stem B content significantly increased in SX from 10.92 mg/Kg to 11.74 mg/Kg and from 12.99 mg/Kg to 25.58 mg/Kg in YD (Fig. 4a). The leaf B content increased significantly in SX from 30.84 mg/Kg to 97.21 mg/Kg and from 66.83 mg/Kg to 136.04 mg/Kg in YD (Fig. 4a). Among these, the amount of B was highest in the YD leaves (136.04 mg/Kg). Although to start with, SX had higher B in the root, but with B spray, YD had more B in its root. Also, before treatment, the B content of YD leaves (66.83 mg/Kg) (Fig. 4a) than SX (30.84 mg/Kg). Before treatment, the B content of the SX female flower (16.62 mg/Kg) SX was higher than the male (13.30 mg/Kg) (Fig. 4b), but after B spray, both male and female flowers had similar B content (20.95 mg/Kg and, 21.01 mg/Kg respectively). Before treatment, the B content of YD male (20.44 mg/Kg) and female (20.38 mg/Kg) flower was higher than the SX, and after treatment, the female YD flower (22.64 mg/Kg) had a higher B content than the YD male flower (21.07 mg/Kg) and both of the SX flowers (Fig. 4b). Above results indicates YD has higher B translocation than SX and is more prominent in foliage and female reproductive organs.

### Jasmonic acid content

**Fig. 5:**
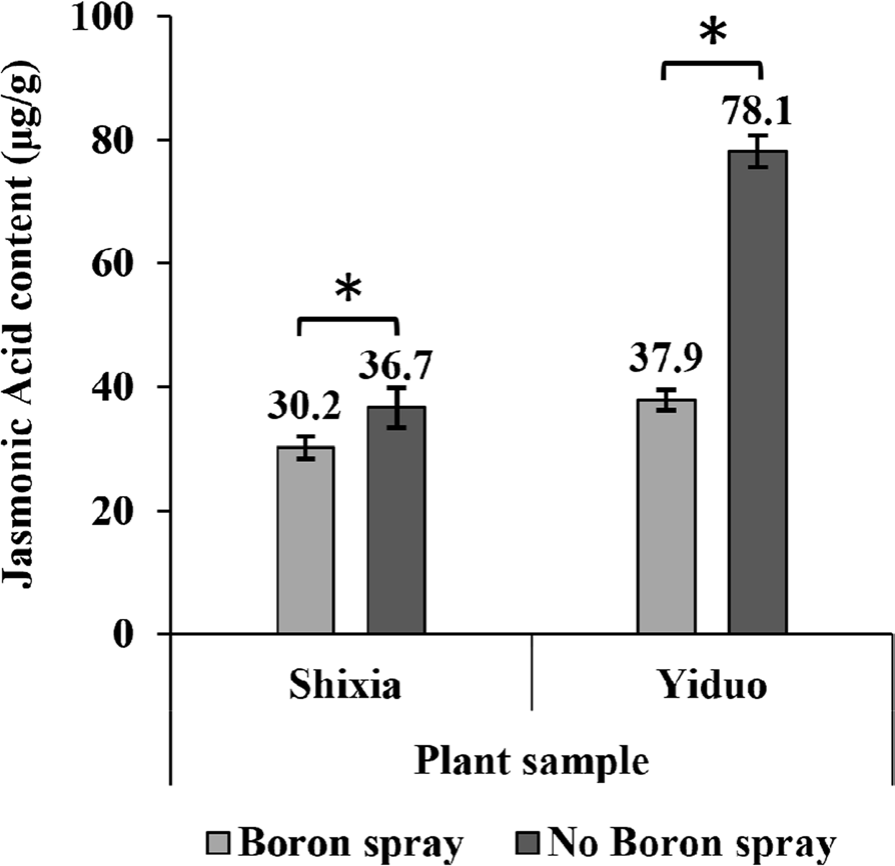
Effect of B spray on JA content of Longan. Foliar B spray increases the JA content of female YD and SX Longan flowers. The Y-axis here shows JA concentrations in µg/g of SX and YD female flower with and without B spray, the bars depict mean ± standard deviation of the biological replicates (*n* = 3), and the asterisks indicate significant differences between the B unsprayed and sprayed plants of the same variety at *p* ≤ 0.05 (*) in the students *t-*test

The YD and the SX Longan female flower’s JA concentrations significantly rose following foliar B spray, with the former showing a more noticeable change. After foliar B spray, the JA content of the SX increased from 30.2 μg/g to 36.7 μg/g, whereas in the YD, it increased from 37.9 μg/g to 78.1 μg/g (Fig. 5).

### Gene expression analysis

We found five candidate genes of the BOR gene family (Additional file 1: Table 1) and thirty-three candidate genes of the NIP gene family (Additional file 2: Table 2). The cis-acting element of the genes was identified using Gene Structure Display Server (GSDS) and visualized in Additional file 3: Fig. 1. Eight different cis-acting elements including salicylic acid responsiveness, core promoter element, stress responsiveness, light responsive element, MeJA-responsiveness, regulatory element, zein metabolism regulation, flavonoid biosynthetic genes regulator, promoter and enhancer regions, anaerobic induction, salicylic acid responsiveness, etc. were identified in the BOR genes promoters (Additional file 3: Fig. 1a). Fourteen different cis-acting elements such as meristem expression, light responsive element, conserved DNA module array, drought-inducibility, MeJA-responsiveness, abscisic acid responsiveness, anaerobic induction, gibberellin-responsiveness cell cycle regulation, core promoter element, etc. were found in the NIP genes promoters (Additional file 3: Fig. 1b). The gene extraction and alignment using the clustalW revealed six BOR and nine NIP domains, respectively (Additional file 4: Fig. 2).

Side-by-side comparison of the phylogenetic analysis (Additional file 5: Fig. 3a and d), the conserved motif (Additional file 5: Fig. 3b and e) and the exon/intron organization (Additional file 5: Fig. 3c and f) of both the BOR and the NIP genes revealed sequence similarity among members of the same phylogenetic domain; however, the distribution of their coding sequences was unique. Ten conserved BOR (Additional file 5: Fig. 3b) and NIP (Additional file 5: Fig. 3e) gene family regions were identified, of which motifs 1, 2, and 3 were shared among all five BOR genes of the gene family, and only motif one was shared among all members of the NIP family (Additional file 6: Table 3).

**Fig. 6:**
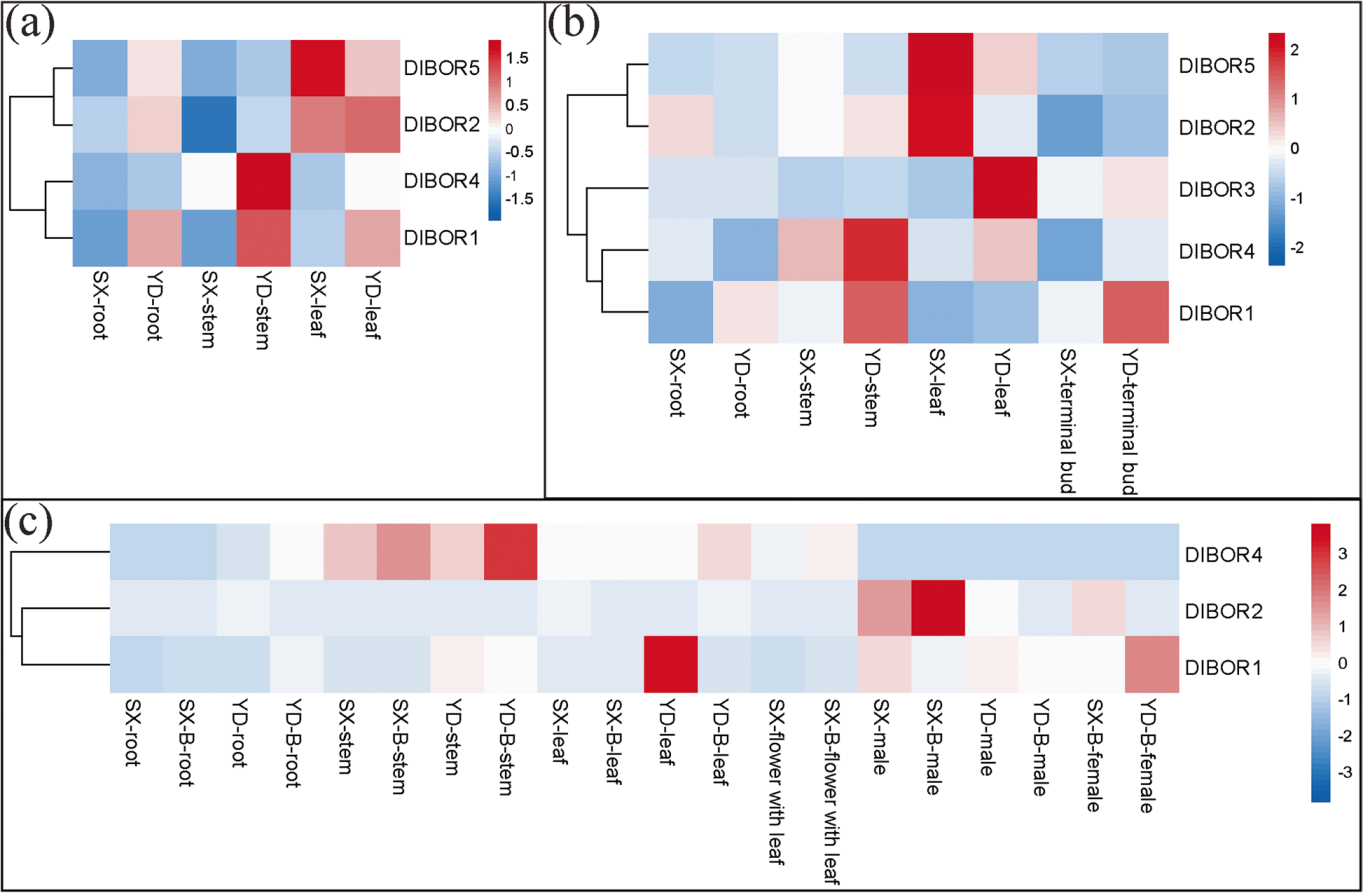
Heatmap-based hierarchical cluster analysis of BOR gene expression in Longan at different stages of B treatment. Longan tissue samples were collected at the beginning of the experiment (**a**), before starting B treatment (**b**), and after one month of B treatment (**c**). Z-score refers to high (red) and low (blue) gene expression using normalized values compared to the mean of total sequencing reads

**Fig. 7:**
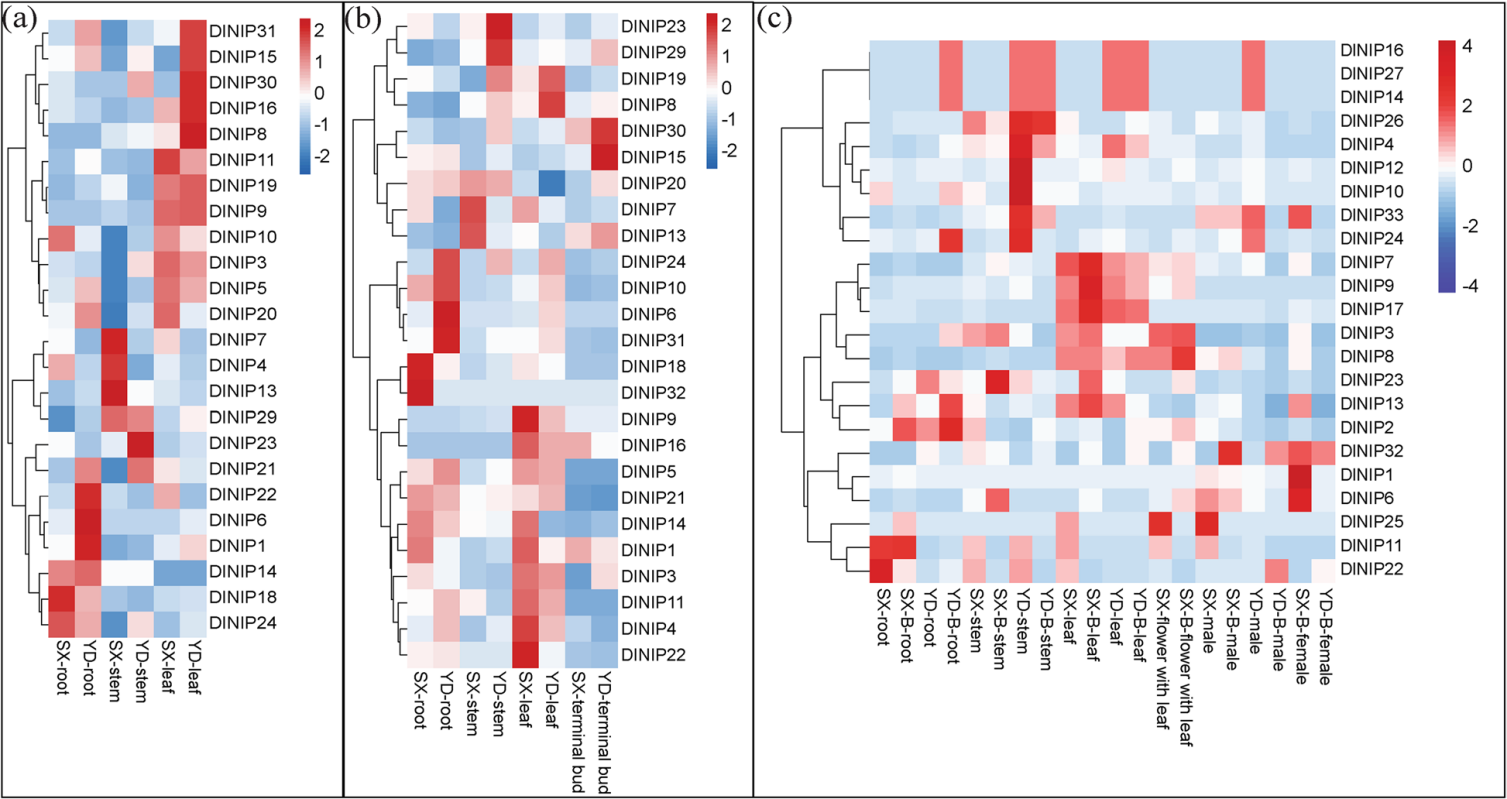
Heatmap-based hierarchical cluster analysis of NIP gene expression in Longan at different stages of B treatment. Longan tissue samples were collected at the beginning of the experiment (**a**), before starting B treatment (**b**), and after one month of B treatment (**c**). Z-score refers to high (red) and low (blue) gene expression using normalized values compared to the mean of total sequencing reads

Knowing the spatiotemporal expression of these genes was also essential to understand their role in B translocation. Heatmap analysis of the BOR (Fig. 6a) and NIP (Fig. 7a) genes expression pattern before B spray revealed that the expression of the NIP genes was more prominent than BOR. DlBOR5 expressed more in the SX leaf, and DlBOR4 expressed more in the YD stem (Fig. 6a).

We also observed high chlorophyll a (Fig. 2h), chlorophyll b (Fig. 2i), and total chlorophyll (Fig. 2j) concentration in the YD shoot. DlNIP8, DlNIP15, DlNIP16, DlNIP30, and DlNIP31 expressed more in the YD leaf; DlNIP23 expressed more in the YD shoot; and DlNIP1, DlNIP6, and DlNIP22 expressed more in the YD root. Whereas DlNIP11 expressed more in the SX leaf, DlNIP4, DlNIP7, and DlNIP13 expressed more in the SX stem, and DlNIP18 expressed more in the SX root (Fig. 7b). It is noteworthy that no two same BOR or NIP gene was upregulated in both SX and YP at the same time. After acclimatization, before B treatment, the appearance of DlBOR3 in the YD leaf, upregulation of DlBOR5 and DlBOR2 in the SX leaf and downregulation of the DlBOR4 in the YD stem were observed (Fig. 6b). DlNIP15 and DlNIP30 were highly expressed in YD bud; DlNIP8 and DlNIP19 were highly expressed in YD leaf; DlNIP4, DlNIP9, and DlNIP22 were highly expressed in SX leaf; DlNIP23 and DlNIP29 were highly expressed in YD stem; DlNIP6 and DlNIP31 were highly expressed in YD root; DlNIP18 and DlNIP32 were highly expressed in SX root (Fig. 7b). After B treatment, most of the BOR genes were absent except for the higher expression of DlBOR2 in the SX male flower, DlBOR1 in the YD leaf, and DlBOR4 in the YD stem (Fig. 6c). With higher B concentration, the expression of BOR family genes gets diminished. A similar pattern was observed for the NIP gene family as we only found higher expression of DlNIP1 in SX female flower; DlNIP9 in SX Leaf; DlNIP22 in SX root; and DlNIP10 was highly expressed in YD’s roots, stems, and leaves. We also observed higher B content in the vegetative organs B untreated YD (Fig. 4), and DlNIP19 might be the reason behind it (Fig. 7b). DlNIP1 is specifically and highly expressed in the female flowers of SX (Fig. 7c), which might be why the B content of female SX flower (Fig. 4b) increased with foliar B spray, but the JA content did not (Fig. 5).

### Subcellular localization

**Fig. 8:**
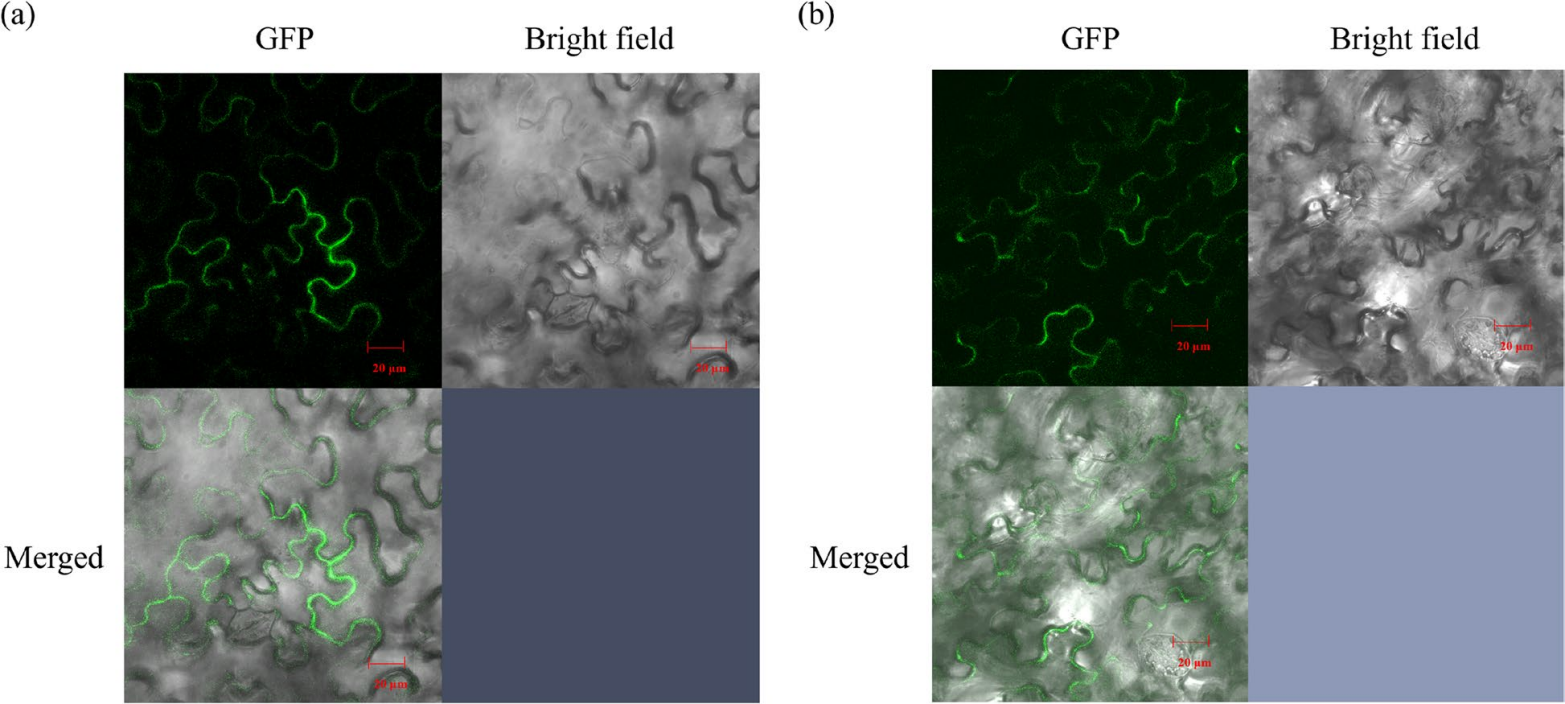
Subcellular localization of GFP tagged DlNIP19 and DlNIP1 in *Nicotiana benthamiana*. The inserted DlNIP19 (**a**) and DlNIP1 (**b**) genes are localized in the cell membrane of the tobacco leaves epidermal cells

The DlNIP19 and DlNIP1 linked pBE-GFP vectors were transfected into tobacco leaf using *Agrobacterium.* The DlNIP19-GFP (Fig. 8a) and the DlNIP1-GFP (Fig. 8b) were observed to express and localize in the extremity of the leaves mesophyll pavement cell, the cell membrane. We also used the sequence of these two DlNIPs to search the WolfPSORT [9] database and found their subcellular localization to be the membrane of the vacuole, Golgi, and endoplasmic reticulum.

### Pollen germination experiment

**Fig. 9:**
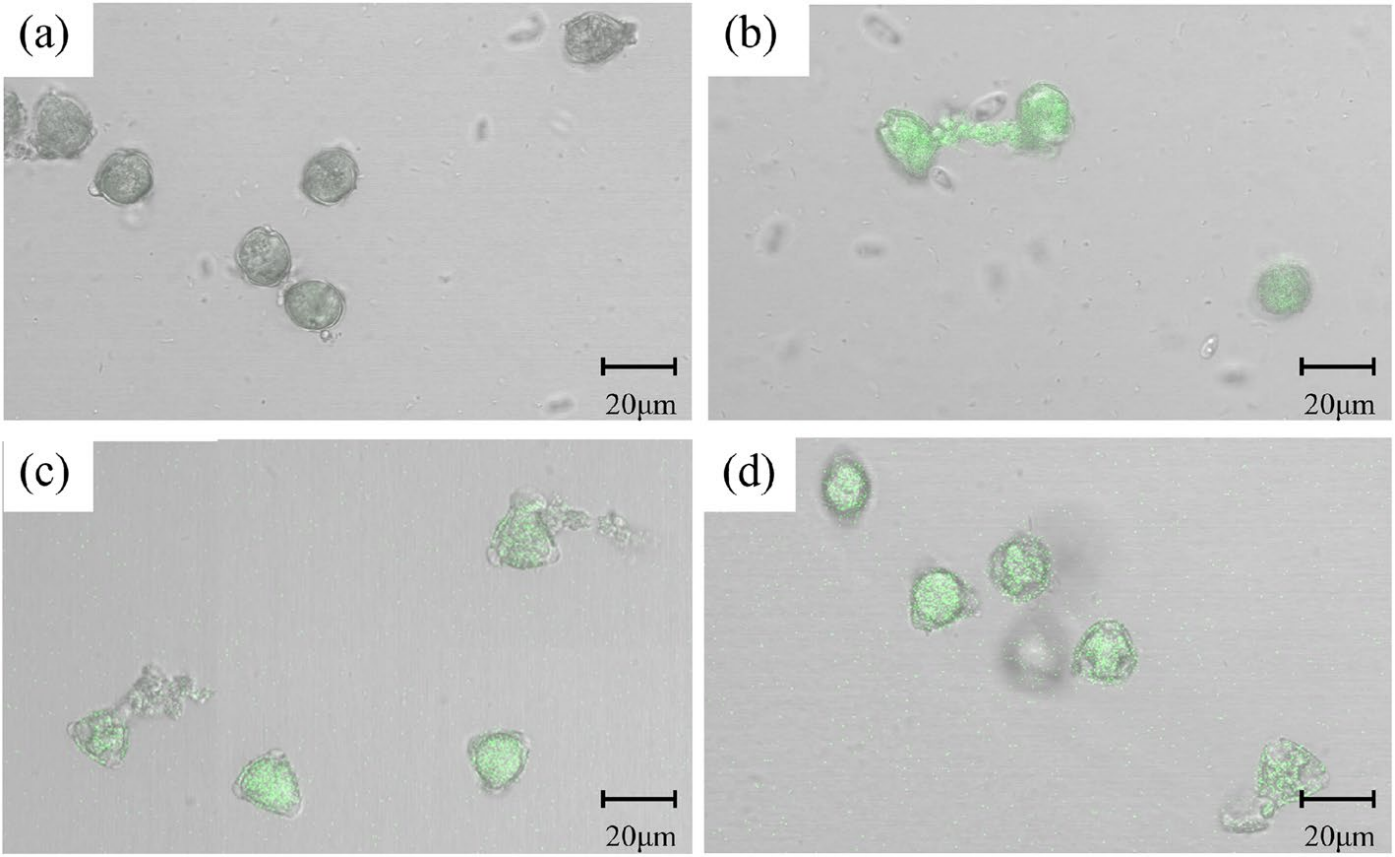
Expression of DlNIP genes. **a** Untransformed pollen. **b** Pollen transformed with empty pBE-GFP plasmid. **c** Pollen transformed with DlNIP19-pBE-GFP plasmid. **d** Pollen transformed with DlNIP1 pBE-GFP plasmid

**Fig. 10:**
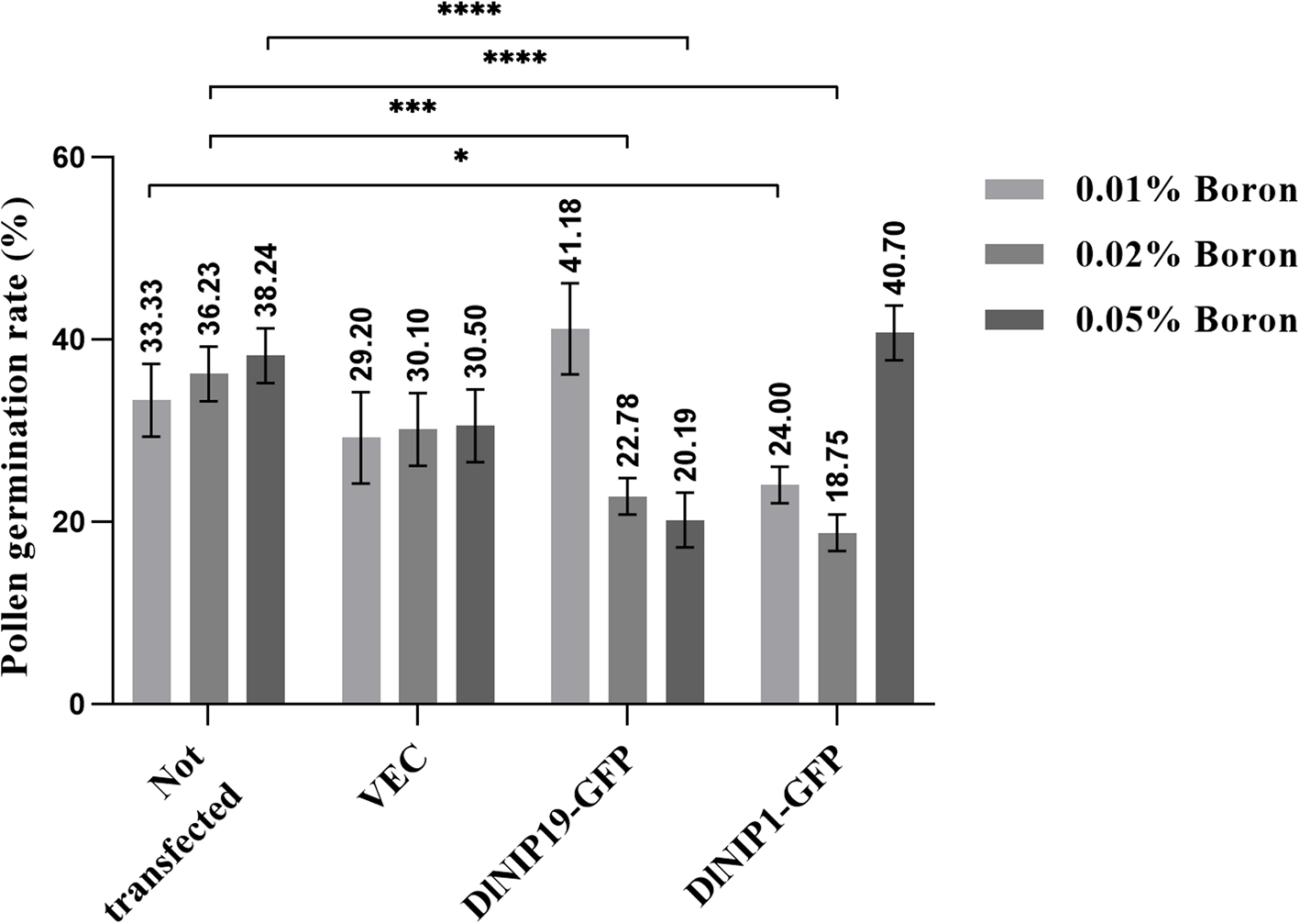
Effect of incrementing B on pollen germination rate. With increasing B concentration in the solid medium, untransformed SX pollen germination gradually increased; the SX DlNIP19-GFP pollen germination decreased while the SX DlNIP-GFP pollen increased. The Y-axis of the chart shows the percentage (%) of germination rate of 0.01%, 0.02%, 0.05% B treated non-transfected (Not-transfected), empty vector-transfected (VEC), DlNIP19-GFP transfected (DlNIP19-GFP), and DlNIP1-GFP transfected (DlNIP1-GFP) pollens. The bars represent the mean ± standard deviation of the biological replicate (*n* = 3) of each group, and the asterisks indicate significant differences between different transfected groups of the same B treatment at * = *p* ≤ 0.05, *** = *p* ≤ 0.001, **** = *p* ≤ 0.0001 in the 2-way ANOVA analysis

**Fig. 11:**
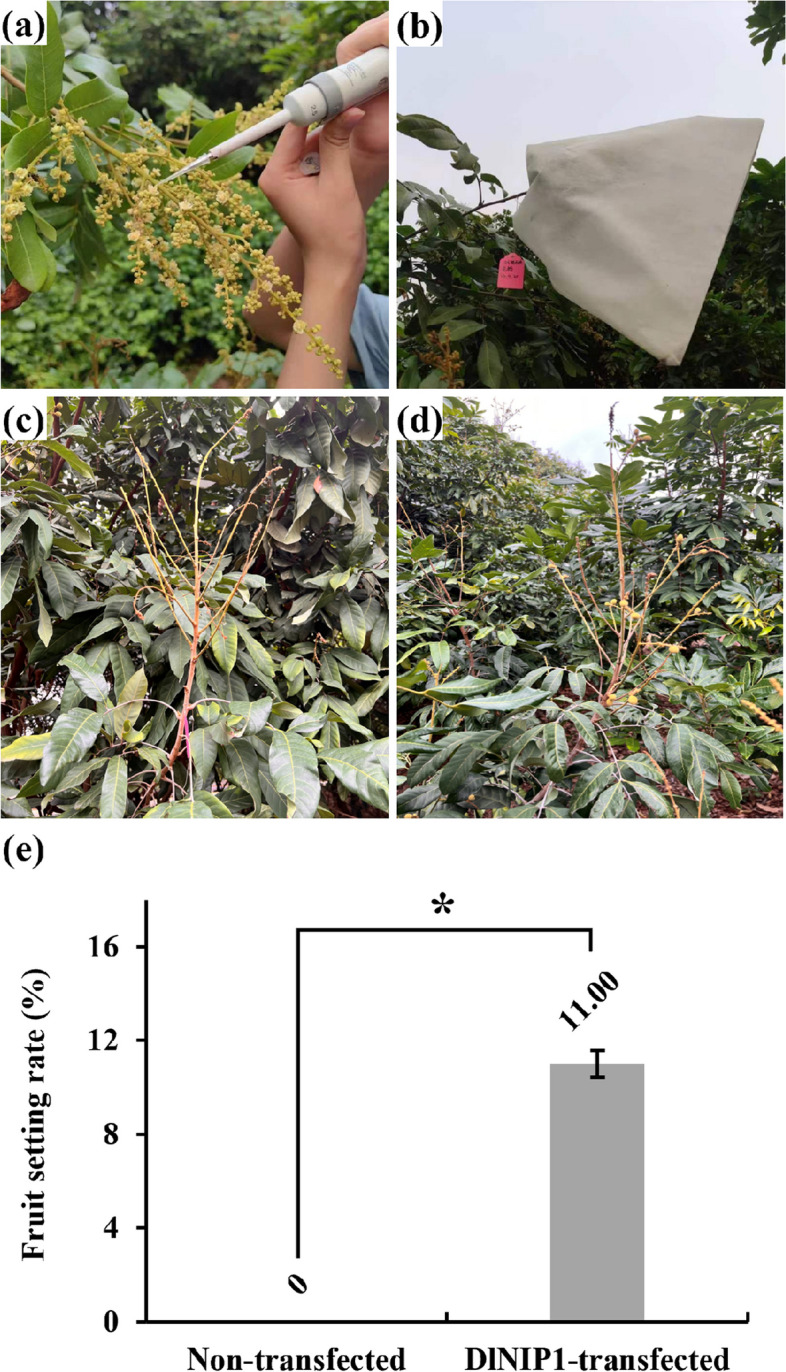
Pollination of YD flowers with transformed SX pollens. **a** YD flowers are inoculated with DlNIP1-transformed SX pollen. **b** Flower panicles are bagged after pollination. **c** YD fruit setting after one month of natural pollination. **d** YD fruit setting after one month of artificial pollination. **e** The fruit setting rate of the DlNIP1 transformed SX pollen inoculated YD panicles. The chart’s Y-axis shows the fruit setting rate of the non-transfected and transfected pollen germinated YD flowers. The bars represent the mean ± standard deviation of the group's biological replicates (*n* = 3) with asterisks indicating significant differences between groups at * = *p* ≤ 0.05 in a student's *t*-test

DlNIPs linked to pBE-GFP were also used to transform SX pollen using magnetic nanocarriers MNPs, and the transformations were confirmed using confocal laser scanning microscopy. GFP expressions were observed in untransformed pollen, and pBE-GFP, DlNIP19-GFP, and DlNIP1-GFP transformed SX pollen (Fig. 9a-d), while the non-transformed pollen showed no fluorescence. Quantitative real-time polymerase chain reaction (RT-PCR) quantified the expression of transferred genes in transgenic pollens (Additional file 7: Fig. 4). We found that compared to untransformed pollen, the DlNIP19-GFP pollen germinates better (41.18%) at low B concentrations (0.01%) but decreased significantly at intermediate (0.02%) and higher (0.05%) concentrations (22.78%, 20.19% respectively) (Fig. 10). In contrast, compared to untransformed pollen, the DlNIP1-GFP pollen germinates better (40.70%) at higher (0.05%) B concentrations but significantly lowered at low (0.01%) and intermediate (0.02%) concentrations (24%, and 18.75% respectively) (Fig. 10). Untransformed and empty vector transformed pollen showed a steady increase in germination with increasing B concentration (Fig. 10). Transgenic DlNIP1-SX and normal SX pollens were used to artificially pollinate YD female flower from 5 panicles of each tree (Fig. 11a) and bagged to prevent contamination (Fig. 11b). DlNIP1-SX pollinated YD Longans (YD♀ × DlNIP1-SX♂) showed increased fruit setting rate (11%) of crossbred (Fig. 11d, e) over the untransformed SX pollinated plants (Fig. 11c).

## Discussion

Cell wall biosynthesis is one of the major factors influencing plant reproduction and growth. The differential expression of related genes, hormones, and the availability of nutrients such as sucrose, starch, etc., affects it [6]. B is the most abundant component of the vascular plant's cell wall (80–90% dry weight), forming borate ester cross-linked rhamnogalacturonan II dimmer essential for cell wall structure and function [8]. We observed thicker cell walls in the mesophyll cells of YD Longan, which were probably due to the higher B content in their leaves (Fig. 4a). Stronger cell walls and thicker vascular bundles (Fig. 1g) allowed YD to attain more significant translocation resulting in increased cellulose (Fig. 2e) and decreased lignification (Fig. 2f). However, cellulose and lignification of SX increased with B treatment. This, along with other changes in the leaf constituents due to B treatment, indicated the presence of a more complex system other than simple diffusion. Shireenet al. [1] also reviewed the effect of B in maintaining various cellular processes and components. They reported decreased carbohydrate metabolism, sugar transport, and photosynthesis due to low B content.

The foliar B spray has increased the fruit-setting rate (Fig. 3) in the SX more than in the YD, probably due to higher JA content in the YD compared to the SX (Fig. 5). Indigenous JA is a plant growth regulator (PGR) associated with abiotic stresses [10]. It has a complex synergistic and antagonistic relationship with other PGRs in managing stress. Under low B conditions, the JA biosynthesis gene upregulates and inhibits the growth in *A. thaliana* [11]. B is also known to influence pollen tube growth and pollination in hybrid Longan [6], and applying JA inhibitors such as ibuprofen and B increases the fruit-setting rate [7].

B is present in the soil as boric acid (H_3_BO_3_) or borate; unlike the other vital elements, B percolates as uncharged molecules rather than ions [1]. The plant has three types of boric acid transport mechanisms. The first is the simple diffusion of uncharged boric acid across lipid bilayers. The second is facilitated diffusion of boric acid via MIP, such as NIP. The third is the export of borate via BOR [3]. Passive diffusion and NIP transport are used when B is present in sufficient amounts, but BOR transporters are used when B is deficient [4]. It is shown that to maintain tissue B homeostasis, the gene expression that codes for these transporters is carefully regulated in response to the level of B present in the environment [2]. Our search found five genes of the BOR gene family (Additional file 3: Fig. 1a) and thirty-three genes of the NIP gene family (Additional file 3: Fig. 1b) differentially expressed in Longan. Among the eight cis-acting elements in BOR and fourteen cis-acting elements in NIP, several core promoters, promoter enhancers, stress-related promoters, flavonoid, and plant growth regulators-related promoters were identified. This indicates the BOR and NIP gene’s association with different stress and their role in plant growth and other physiological processes. When aligned with Citrus, Arabidopsis thaliana, and Litchi, we discovered the BOR gene family had six major clusters (Additional file 4: Fig. 2a), and the NIP gene family had nine (Additional file 4: Fig. 2b). However, Ozyigit et al. [4] reported three major BOR1 clusters and three major NIP5;1 cluster. Ten motifs were selected from the five BOR and thirty-three NIP genes to show the evolutionary relationships among the family members (Additional file 5: Fig. 3). However, in a study of NIP5;1, NIP6;1 and BOR1 genes expression among 18 different plants, Ozyigit et al. [4] reported identifying five conserved regions for NIP and BOR gene family.

Heatmap analysis of gene expression at different stages of development showed that after acclimatization, the BOR gene expression increased but decreased with increasing B concentration (Fig. 6). In their review, Miwa et al. [2] emphasized the function of BOR1 in B acquisition by root and translocation into the shoot in *Oryza sativa*. The expression of BOR1 in *A. thaliana* is controlled post-transcriptionally, where it is accumulated in the plasma membrane under low B conditions. However, in high B conditions, it is incorporated into the endosome, transported to the vacuole and degraded [12]. According to Miwa et al. [13], plants that overexpress BOR4 are better able to extend their leaves and store more chlorophyll in the shoot tissues in environments with high B availability. This coincides with our observation of stable chlorophyll concentration (Fig. 2h, i, and j) and over-expressed BOR4 in the YD (Fig. 6b).

After B spray, DlNIP19 expressed most in the YD vegetative organs, and DlNIP1 expressed most in SX females (Fig. 7c), and when injected in tobacco leaf using *A. tumefacient*, both were found to localize in the cell walls. Since DlNIP1 expressed more in female flowers, the DlNIP1 transformed SX pollen germinated better at increasing B concentration than the DlNIP19 transformed SX pollen (Fig. 10). In the NIP gene family tree, DlNIP1 belongs to the NIP7 cluster, and DlNIP19 belongs to the NIP4 cluster (Additional file 4: Fig. 2). Genes from the NIP4 cluster were reported to be specific to the pollen [14] and NIP7 to the anther [15]. This indicates they have a different role in B transport. In *A. thaliana,* the NIP5;1 facilitates the influx of B into root cells from the soil and the NIP6;1 promoter showed strong expression at nodes of the stem [2]. Ozyigit et al. [4], on the other hand, showed that, in B-deficient conditions, the protein NIP5;1 was localized to the plasma membrane of the root cap and epidermal cells and highly increased, whereas the protein NIP6;1 was implicated in B distribution but in the shoots. JA has been reported to influence pollen germination negatively [6], which could be the reason behind the lower seed-setting rate in the female YD-containing crossbreed. Higher germination of DlNIP1 in high B also indicates its role in female flowers is crucial for germination (Fig. 10). Conversely, a lower DlNIP19 germination rate suggests it is more suited for managing high B in the vegetative organs.

Due to UCI, the seed setting rate between the YD♀ × SX♂ and the SX♀ × YD♂ were 0% and 22.4% [6]. We found that the YD pollinated using DlNIP1 transformed SX pollen had a better seed setting rate than the normal YD♀ × SX♂. It demonstrates the possibility of overcoming the UCI and making YD♀ × SX♂ Longan capable of harbouring the benefiting traits of both breeds.

## Conclusion

B transporter BOR and NIP gene family plays a vital role in regulating longan metabolism, pollination, and growth regulators during low, normal, and high B conditions. Our study suggests different genes of these two gene families have activated in SX and YD Longan cultivars at different stages and B stresses during their cultivation, affecting their constituents and growth regulators differently. We selected two high-expressing NIP genes to study their spatiotemporal expression and found their accumulation in the plasma membrane. This differential expression is one of the reasons for the UCI between SX and YD. However, using YD♀ × SX♂, we achieved a higher seed setting than the naturally pollinated YD. *In-vitro* germination experiment showed that DlNIP1 transformed pollen germinates more than the DlNIP19 transformed pollen in an increasing B level. We hope this study will inspire further research in understanding the role of these B transporters for the betterment of the development of high-yielding Longan hybrid crops.

## Methodology

### Plant growth, treatments, and tissue collection

The experiment was conducted in Longan Resource Nursery of Guangzhou City, Guangzhou, China. We selected ten YD and ten SX healthy, ten-year-old Longan trees with similar growth for this experiment. The foliar spray of liquid sugar alcohol boron (151 g/L) acquired from Qingdao Arthur Import and Export Trade Co., Ltd. was diluted to 150 mg/L. The solution was sprayed once every two weeks onto five trees from both varieties. Roots, stems (twigs), leaves (final mature leaves), and flowers (male and female) were collected from all the trees at the beginning and the end of the B treatment, with three replicates for each. They were partly dried to measure the total B content. The others were frozen in liquid nitrogen and stored at -70 °C to detect gene expression. We collected female flowers from B-treated and untreated plants to measure the JA content and reveal the difference in floral organ PGR content under different B conditions. We collected leaves for optical and electronic microscopic analysis to discover the cell wall structure under various B conditions.

### Leaf phenotyping

Mature YD and SX leaves were collected 130 days after B treatment. The anatomical characteristics of cells and vessels were evaluated in samples of mature leaves. Tissue samples (20mm^2^) observed under light microscopy (LM) were collected from the middle third of the leaf between 8:00 and 9:00 am. Tissue samples selected for LM were then fixed, sliced, and stained for semithin section LM and TEM following the methodology described by Mesquita et al. [16]. The concentration of sucrose, glucose, Quina cortisol, fructose, cellulose, lignin, starch, chlorophyll a, chlorophyll b, total chlorophyll, and carotenoids in the frozen leaf samples from B untreated and B treated plants were analyzed using tandem mass spectrometry method described by C Sousa [17].

Leaf phenotypes were visualized as mean ± standard in a bar diagram. The significant difference was calculated using the student's *t-*test between the two varieties.

### Fruit-setting rate

The fruit-setting rate was calculated following the methods described by De Silva et al. [18] and Wang et al. [7]. First, we tagged five racemes on each tree before flowering and counted the average number of flowers on each during peak anthesis. After 50 days, the number of mature fruits remaining on a raceme was counted, and the fruit-setting rate was calculated using the following equation.

$$Fruit\;setting\;\left(\%\right)=\frac{Number\;of\;fruit\;remaining\;on\;the\;raceme}{Number\;of\;floweres\;on\;the\;raceme\;at\;peak\;anthesis}\times100\%$$

The fruit-setting rate was visualized as mean ± standard deviation in a bar diagram. The significant difference was calculated using the student’s *t*-test between boron untreated and treated samples.

### Total Boron content

The total B content of the dried plant tissue samples collected before and after B treatment was performed using the multi-collector inductively coupled plasma mass spectrometers with the sample preparation and analysis method adopted from Xiao et al. [19]. Total boron content was visualized as mean ± standard deviation in a bar diagram. The significance of untreated and treated samples, with the samples from the beginning, was calculated using 2-way ANOVA analysis.

### Jasmonic acid content

We collected female flowers from all the treated and untreated plants to evaluate the difference in B foliar spray on JA content. JA content was determined with a triple quadrupole liquid chromatography-mass spectrophotometry system following the method described by Wang et al. [6] for sample preparation and analysis. JA content was visualized as mean ± standard in a bar diagram. The significant difference was calculated using the student’s *t-*test between boron untreated and treated samples.

### Gene expression analysis

#### RNA extraction and gene expression analysis

Total RNA was extracted from the frozen samples using Trizol method as described by Xie et al.> [20]. We used Agarose gel electrophoresis and Nanodrop ND-1000 spectrophotometer for RNA analysis and quantification. According to the manufacturer's instructions, the HiScript® II 1st Strand cDNA Synthesis Kit (Vazyme) was used to create the first strand of cDNA from DNA-free RNA. The Roche Lightcyler® 480 apparatus was used to perform the quantitative RT-PCR utilizing SYBR Green chemistry. The Longan GAPDH gene was used as an internal control. The reaction was conducted as follows: 95 °C for 30 s, then 40 cycles of 95 °C for 10 s, followed by 30 s of 60 °C. The data from real-time PCR amplification was examined using the 2^ − △△CT^ technique, and each experiment was carried out in biological triplicates [20].

#### Identification of BOR and NIP gene family in the Longan genome

The Hidden Markov Model (HMM) analysis was used for searching Longan BOR family candidate genes. We downloaded the HMM profile of BOR (PF00955) [21], and genes with an e-Value < 0.001 were selected as candidate gene family genes. To identify Longan NIP candidates, the protein sequence file of the *Arabidopsis* NIP gene family was downloaded and using the blastp suite (https://blast.ncbi.nlm.nih.gov/Blast.cgi?PAGE=Proteins), all protein sequences in the genome were searched, and screened the genes with e-Value < 0.001 to get the preliminary identification results. The homoeopathic elements of the upstream sequences of the gene family members were analyzed using the online website of plantcare [22] (http://bioinformatics.psb.ugent.be/webtools/plantcare/html/). Then, we use the online software GSDS [23] (http://gsds.cbi.pku.edu.cn/) to visualize cis-acting elements.

#### BOR and NIP gene family phylogenetic tree analysis

According to the ID information of the identified BOR and NIP gene family genes, the domain sequences of Longan (DI), Citrus (Cs), *A. thaliana* (At), and Litchi (Lc) were extracted. Multiple sequence alignment was performed with clustalW [24]. Then, BOR and NIP gene family trees were constructed using the NJ (neighbour joining) method and visualized using MEGA11 [25] (https://www.megasoftware.net/).

#### Gene family gene motif, structure, and evaluation analysis

To understand the gene structure and the evolutionary relationship between the genes of the BOR and the genes of the NIP gene family, we used the MEME [26] (http://meme-suite.org/) software to identify their motifs. Then, we used the GSDS [23] (http://gsds.gao-lab.org/) database to illustrate the structure and evolution of the BOR and NIP gene family genes.

#### Heat map analysis of gene expression

Heat map-based hierarchical cluster analysis of genes of the BOR and NIP gene families was performed using the heatmaps [27] package of the R (https://www.r-project.org/) statistical analysis software.

### Vector plasmid construction

In light of previous analysis, two genes most expressed in Longan tissue and female flower were selected for further study, and each gene was linked with a pBE-GFP plasmid to obtain two gene overexpression vector plasmids using ClonExpress IIOne Step Cloning Kit (Vazyme, China). These plasmids were then used to transform *Agrobacterium* by electroporation [28].

### Subcellular localization analysis

We injected each transformed bacterium into the lower epidermis of the 1-month-old tobacco plant’s leaf to study subcellular localization. After 48 h of infiltration, GFP fluorescence was seen and recorded by a laser confocal microscope (Leica, Germany).

### Polen germination experiment

DlNIPs were linked with pBE-GFP plasmid to obtain gene overexpression vector plasmid. The nano gene carrier system was constructed by linking MNPs (purchased from Nanjing Dongna Biological Co., Ltd.) to plasmid deoxyribonucleic acid (DNA) to form MNP/DNA complexes [28]. An external magnetic field was applied to widen the pollen pore and allow the MNPs to introduce the DNA into the SX pollen [29]. Hitachi S-3400N-II SEM was used for observation, and quantitative RT-PCR analysis was performed to quantify the expression of transferred genes in transgenic pollens. The GFP signal was observed with a Zeiss LSM710 laser confocal scanning microscope in the pollen liquid medium, and the Longan pollen overexpressing genes were obtained.

The untransformed pollen and pollen transformed with only pBE-GFP were cultured in pollen culture media. Then, both NIP-transformed and untransformed pollens were subcultured in solid media containing 0.01%, 0.02%, and 0.05% (w/v) H_3_BO_3_ to a concentration of no more than 30 pollen grains per 0.02 ml of medium and the cultures were adjusted to pH 6.4 and incubated at 28 °C in light culture for 10 h. Pollen grains were considered germinated when the pollen tube length was more significant than the diameter of the pollen grain. Pollen germination rate was determined by calculating the percentage of germination of pollen grains in at least ten aliquots per treatment. All experiments were performed in triplicate, and all observations were made with a Zeiss LSM710 laser confocal scanning microscope.

Also, transgenic DlNIP was used to pollinate the YD stigmas to produce YD♀ × SX♂ cross hybrid since it was reported that only this combination of reciprocal combination works in Longan due to UCI [6].

### Supplementary Information

**Additional file 1: Table 1. **Features of BOR genes identified in Longan.

**Additional file 2: Table 2.** Features of NIP genes identified in Longan.

**Additional file 3: Fig. 1.** Genes of BOR and NIP gene family. Cis-acting element of genes of BOR (a) and NIP (b) gene families.

**Additional file 4: Fig. 2.** Phylogenetic tree of the boron transporter gene families. (a) Six subclasses of BOR gene families were identified from 21 BOR genes. (b) Nine subclasses of NIP gene families were identified from 102 NIP genes.

**Additional file 5: Fig. 3.** Phylogenetic relationship, gene structure and conserved motif analysis of the boron transporter gene families. Phylogenetic tree of BOR (a) and NIP (d) proteins. Distributions of conserved motifs in BOR (b) and NIP (e) genes where ten putative motifs are indicated in different coloured boxes. Exon/intron organization of BOR (c) and NIP (f) genes where the green boxes represent exons and the grey lines represent introns.

**Additional file 6: Table 3.** Most conserved motifs in BOR and NIP homologs in SX and YD.

**Additional file 7: Fig. 4.** Gel electrophoresis of PCR products of transferred genes from transgenic pollens. Transfection of NIP19-GFP and NIP1-GFP pollens was confirmed by amplifying three biological repeats from each and resolving them on agarose gel electrophoresis.

## Data Availability

We summarized the datasets used in this manuscript and presented them as supporting information for publication. The corresponding author will make any other relevant information available upon reasonable request.

## Abbreviations

NIP: Nodulin 26-like intrinsic proteins
BOR: Borate transporters
SX: Shixia
YD: Yiduo
GFP: Green fluorescent protein
MIP: Major intrinsic proteins
JA: Jasmonic acid
UCI: Unilateral cross incompatibility
TEM: Transmission electron microscopy
GSDS: Gene Structure Display Server
SEM: Scanning electron microscopy
RT-PCR: Real time polymerase chain reaction
PGR: Plant growth regulator
LM: Light microscopy
HMM: Hidden Markov Model
MNP: Magnetic nanocarriers
DNA: Deoxyribonucleic acid
