# Supplementary material for: Boron homeostasis affects Longan yield: a study of NIP and BOR boron transporter of two cultivars
Source: BMC Plant Biol. 2024 Jan 2;24:9. doi: 10.1186/s12870-023-04689-8 (PMC10759464; doi:10.1186/s12870-023-04689-8)
Supplement: Supplementary file 1 — Additional file 1: Table 1. Features of BOR genes identified in Longan. [file 12870_2023_4689_MOESM1_ESM.docx]

**Table. 1: Features of BOR genes identified in Longan.**

| **Name** | **Gene ID** | **mRNA ID** | **Chr.** | **Protein (AA)** | **CDS (bp)** | **Exons** | **MW (MV)** | **pI** |
| --- | --- | --- | --- | --- | --- | --- | --- | --- |
| ***DlBOR1*** | D.long000082 | D.long000082.01 | 1 | 709 | 2130 | 12 | 79067.6 | 9.05 |
| ***DlBOR2*** | D.long003518 | D.long003518.01 | 1 | 654 | 2028 | 11 | 73773.5 | 7.34 |
| ***DlBOR3*** | D.long012393 | D.long012393.01 | 2 | 642 | 2001 | 13 | 71380.6 | 8.47 |
| ***DlBOR4*** | D.long006193 | D.long006193.01 | 4 | 684 | 2133 | 12 | 76081.1 | 8.46 |
| ***DlBOR5*** | D.long008830 | D.long008830.01 | 13 | 720 | 2163 | 12 | 80342.8 | 9.20 |
