# Supplementary material for: Boron homeostasis affects Longan yield: a study of NIP and BOR boron transporter of two cultivars
Source: BMC Plant Biol. 2024 Jan 2;24:9. doi: 10.1186/s12870-023-04689-8 (PMC10759464; doi:10.1186/s12870-023-04689-8)
Supplement: Supplementary file 2 — Additional file 2: Table 2. Features of NIP genes identified in Longan. [file 12870_2023_4689_MOESM2_ESM.docx]

**Table. 2: Features of NIP genes identified in Longan.**

| **Name** | **Gene ID** | **mRNA ID** | **Chr.** | **Protein (AA)** | **CDS (bp)** | **Exons** | **MW (MV)** | **pI** |
| --- | --- | --- | --- | --- | --- | --- | --- | --- |
| ***DlNIP1*** | D.long000493 | D.long000493.01 | 1 | 299 | 900 | 5 | 79067.6 | 9.05 |
| ***DlNIP2*** | D.long002345 | D.long002345.01 | 1 | 334 | 1017 | 9 | 73773.5 | 7.34 |
| ***DlNIP3*** | D.long003639 | D.long003639.01 | 1 | 244 | 735 | 3 | 31685.6 | 6.66 |
| ***DlNIP4*** | D.long012222 | D.long012222.01 | 2 | 162 | 494 | 3 | 38349.8 | 10.17 |
| ***DlNIP5*** | D.long012223 | D.long012223.01 | 2 | 302 | 938 | 5 | 25894.1 | 6.50 |
| ***DlNIP6*** | D.long012569 | D.long012569.01 | 2 | 247 | 744 | 3 | 18468.8 | 9.13 |
| ***DlNIP7*** | D.long004908 | D.long004908.01 | 4 | 245 | 759 | 3 | 33433.6 | 8.59 |
| ***DlNIP8*** | D.long005339 | D.long005339.01 | 4 | 282 | 849 | 4 | 25093 | 5.44 |
| ***DlNIP9*** | D.long005347 | D.long005347.01 | 4 | 285 | 858 | 4 | 27205.1 | 7.30 |
| ***DlNIP10*** | D.long015627 | D.long015627.01 | 5 | 125 | 378 | 4 | 29862.6 | 9.32 |
| ***DlNIP11*** | D.long024532 | D.long024532.01 | 6 | 223 | 672 | 7 | 30371.4 | 9.28 |
| ***DlNIP12*** | D.long024534 | D.long024534.01 | 6 | 258 | 836 | 5 | 13965 | 8.69 |
| ***DlNIP13*** | D.long024535 | D.long024535.01 | 6 | 259 | 815 | 5 | 23991.2 | 7.67 |
| ***DlNIP14*** | D.long016484 | D.long016484.01 | 7 | 268 | 854 | 5 | 29239.1 | 10.19 |
| ***DlNIP15*** | D.long016485 | D.long016485.01 | 7 | 243 | 785 | 5 | 29334.9 | 9.68 |
| ***DlNIP16*** | D.long016486 | D.long016486.01 | 7 | 255 | 815 | 5 | 30677.5 | 9.96 |
| ***DlNIP17*** | D.long023649 | D.long023649.01 | 9 | 308 | 927 | 3 | 27730.3 | 10.10 |
| ***DlNIP18*** | D.long023650 | D.long023650.01 | 9 | 325 | 978 | 2 | 29031.6 | 9.59 |
| ***DlNIP19*** | D.long035517 | D.long035517.01 | 10 | 275 | 828 | 5 | 32964.4 | 6.99 |
| ***DlNIP20*** | D.long035849 | D.long035849.01 | 10 | 183 | 552 | 3 | 35486 | 6.38 |
| ***DlNIP21*** | D.long036743 | D.long036743.01 | 10 | 245 | 774 | 3 | 29242.8 | 8.63 |
| ***DlNIP22*** | D.long027352 | D.long027352.01 | 11 | 261 | 786 | 7 | 19754.8 | 6.85 |
| ***DlNIP23*** | D.long007604 | D.long007604.01 | 13 | 286 | 861 | 4 | 26833.5 | 9.60 |
| ***DlNIP24*** | D.long007743 | D.long007743.01 | 13 | 250 | 753 | 3 | 28080.5 | 4.62 |
| ***DlNIP25*** | D.long009174 | D.long009174.01 | 13 | 243 | 750 | 3 | 30777.4 | 8.18 |
| ***DlNIP26*** | D.long030394 | D.long030394.01 | 14 | 282 | 902 | 4 | 25512.2 | 5.44 |
| ***DlNIP27*** | D.long033439 | D.long033439.01 | 15 | 276 | 881 | 5 | 25845 | 8.23 |
| ***DlNIP28*** | D.long033444 | D.long033444.01 | 15 | 276 | 881 | 5 | 32168.1 | 11.55 |
| ***DlNIP29*** | D.long033450 | D.long033450.01 | 15 | 276 | 881 | 5 | 30671.6 | 8.78 |
| ***DlNIP30*** | D.long033456 | D.long033456.01 | 15 | 275 | 881 | 5 | 30716.6 | 9.14 |
| ***DlNIP31*** | D.long033465 | D.long033465.01 | 15 | 225 | 692 | 5 | 30724.6 | 9.16 |
| ***DlNIP32*** | D.long034318 | D.long034318.01 | 15 | 251 | 756 | 2 | 30459.2 | 8.80 |
| ***DlNIP33*** | D.long034406 | D.long034406.01 | 15 | 287 | 864 | 4 | 25009.5 | 7.18 |
