# Supplementary figures and images for: Boron homeostasis affects Longan yield: a study of NIP and BOR boron transporter of two cultivars
Source: BMC Plant Biol. 2024 Jan 2;24:9. doi: 10.1186/s12870-023-04689-8 (PMC10759464; doi:10.1186/s12870-023-04689-8)

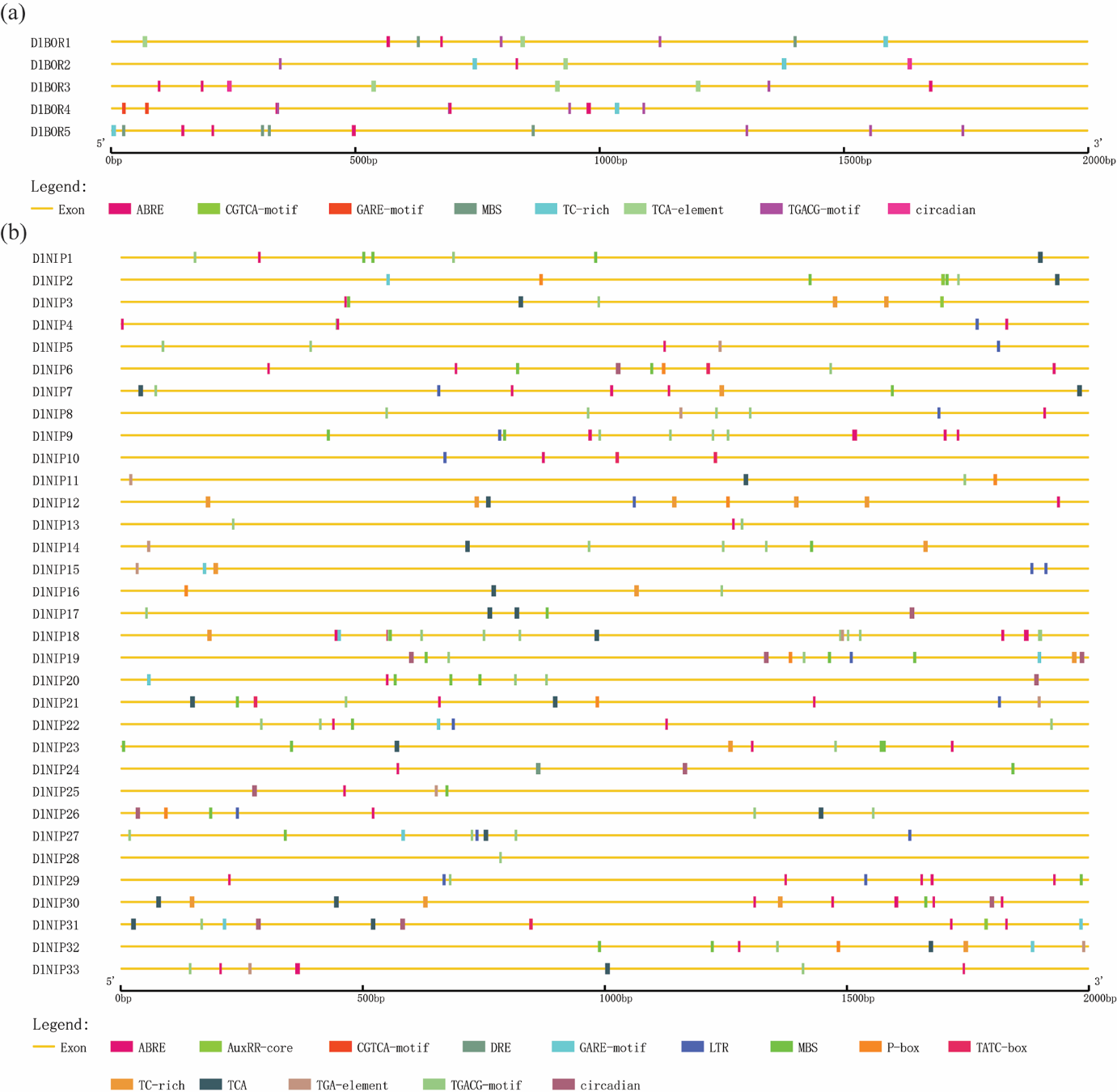


**Fig. 1**: **Genes of BOR and NIP gene family.** Cis-acting element of genes of BOR **(a)** and NIP **(b)** gene families.

Supplement: Supplementary file 3 — Additional file 3: Fig. 1. Genes of BOR and NIP gene family. Cis-acting element of genes of BOR (a) and NIP (b) gene families. [file 12870_2023_4689_MOESM3_ESM.docx]
