# Supplementary material for: Boron homeostasis affects Longan yield: a study of NIP and BOR boron transporter of two cultivars
Source: BMC Plant Biol. 2024 Jan 2;24:9. doi: 10.1186/s12870-023-04689-8 (PMC10759464; doi:10.1186/s12870-023-04689-8)
Supplement: Supplementary file 4 — Additional file 4: Fig. 2. Phylogenetic tree of the boron transporter gene families. (a) Six subclasses of BOR gene families were identified from 21 BOR genes. (b) Nine subclasses of NIP gene families were identified from 102 NIP genes. [file 12870_2023_4689_MOESM4_ESM.docx]

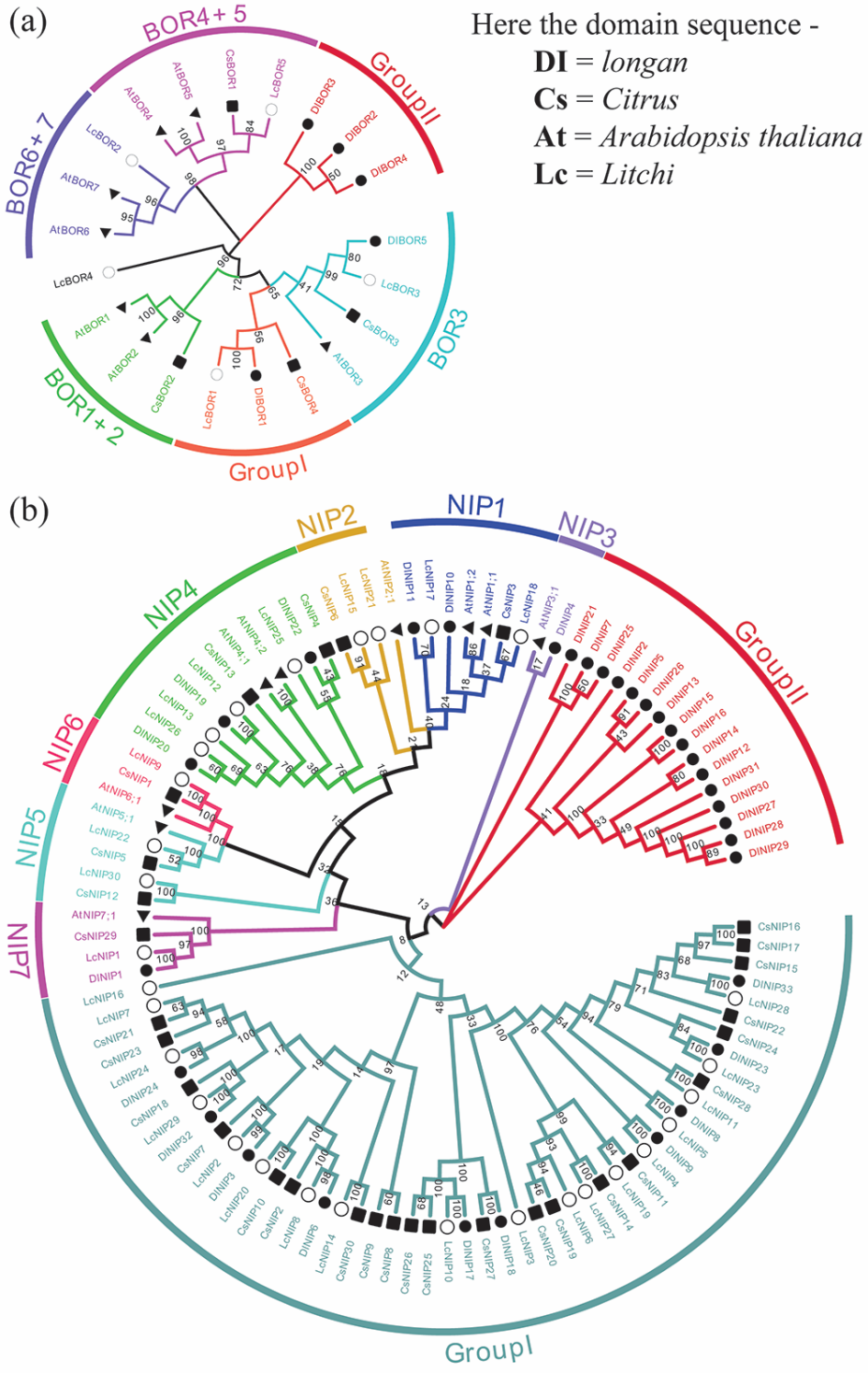


**Fig. 2**: **Phylogenetic tree of the boron transporter gene families.** **(a)** Six subclasses of BOR gene families were identified from 21 BOR genes. **(b)** Nine subclasses of NIP gene families were identified from 102 NIP genes.
