# Supplementary material for: Boron homeostasis affects Longan yield: a study of NIP and BOR boron transporter of two cultivars
Source: BMC Plant Biol. 2024 Jan 2;24:9. doi: 10.1186/s12870-023-04689-8 (PMC10759464; doi:10.1186/s12870-023-04689-8)
Supplement: Supplementary file 5 — Additional file 5: Fig. 3. Phylogenetic relationship, gene structure and conserved motif analysis of the boron transporter gene families. Phylogenetic tree of BOR (a) and NIP (d) proteins. Distributions of conserved motifs in BOR (b) and NIP (e) genes where ten putative motifs are indicated in different coloured boxes. Exon/intron organization of BOR (c) and NIP (f) genes where the green boxes represent exons and the grey lines represent introns. [file 12870_2023_4689_MOESM5_ESM.docx]

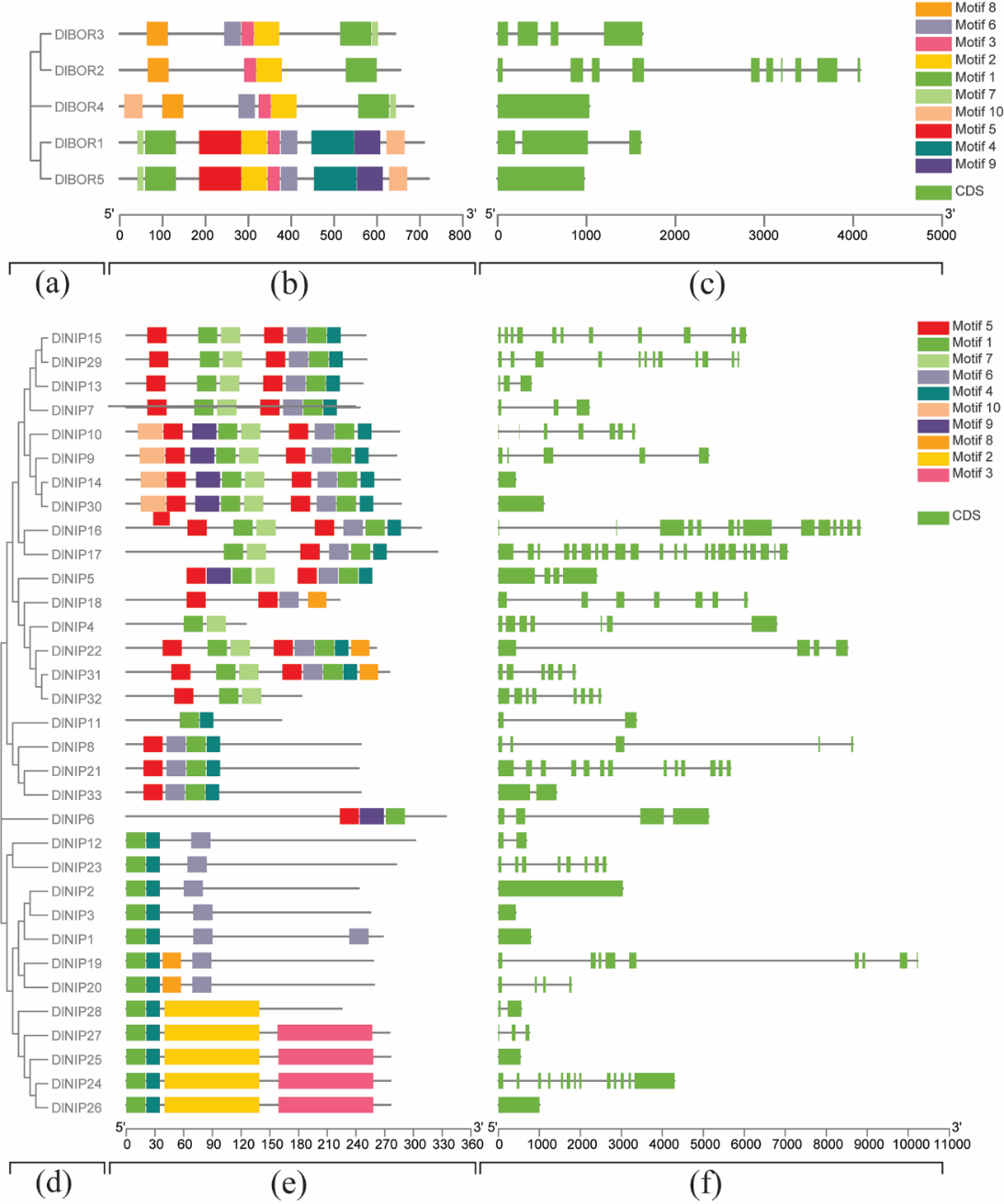


**Fig. 3: Phylogenetic relationship, gene structure and conserved motif analysis of the boron transporter gene families.** Phylogenetic tree of BOR **(a)** and NIP **(d)** proteins. Distributions of conserved motifs in BOR **(b)** and NIP **(e)** genes where ten putative motifs are indicated in different coloured boxes. Exon/intron organization of BOR **(c)** and NIP **(f)** genes where the green boxes represent exons and the grey lines represent introns.
