# Supplementary material for: Boron homeostasis affects Longan yield: a study of NIP and BOR boron transporter of two cultivars
Source: BMC Plant Biol. 2024 Jan 2;24:9. doi: 10.1186/s12870-023-04689-8 (PMC10759464; doi:10.1186/s12870-023-04689-8)
Supplement: Supplementary file 6 — Additional file 6: Table 3. Most conserved motifs in BOR and NIP homologs in SX and YD. [file 12870_2023_4689_MOESM6_ESM.docx]

**Table. 3: Most conserved motifs in BOR and NIP homologs in SX and YD.**

| **Family** | **Motif No** | **Motif Width** | **Motif Sequence** | **E-value** | **Homologous gene/ Sites** |
| --- | --- | --- | --- | --- | --- |
| ***BOR*** | 1 | 73 | DMFNVPVLYIIGAFIPATMIAVLYYFDHSVASQLAQQEEFNLRKPSAFHYDLLLLGFMVI | 1.0e-139 | 5 |
|  | 2 | 60 | DMFNVPVLYIIGAFIPATMIAVLYYFDHSVASQLAQQEEFNLRKPSAFHYDLLLLGFMVI | 4.1e-130 | 5 |
|  | 3 | 29 | CGLIGIPPSNGVIPQSPMHTKSLATLKHQ | 1.6e-070 | 5 |
|  | 4 | 100 | VDDLLPVEVKEQRLSNLLQAIMAGGCVAAMPALKMIPTSVLWGYFAYMAIESLPGNQFWERILLLFTVPSRRYKVLEDYHATFVETVPFKTIAIFTIFQT | 1.3e-037 | 2 |
|  | 5 | 99 | NPNRTALQPSWRFGNGMFALVLSFGLLLTALRSRKARSWRYGSGWLRGFIADYGVPLMVLVWTAVSYIPVNDVPRGIPRRLFSPNPWSHGAYSNWTVIK | 3.4e-030 | 2 |
|  | 6 | 39 | RNRLVATARRSMRKNASLGQLYGNMQEAYQQMQTPLIYQ | 3.8e-026 | 4 |
|  | 7 | 15 | YIFFASAIPVISFGE | 8.6e-018 | 4 |
|  | 8 | 50 | CFICGVSTIQIYCRIHSLPVCIFPGMFRYYMDSYSWNLVSIAILSTHQYK | 6.4e-017 | 3 |
|  | 9 | 61 | AYLFLCFGITWIPIAGVLFPLMIMLLVPVRQYVLPKFFKGAHLSDLDAAEYEEAPAIAYNM | 1.8e-016 | 2 |
|  | 10 | 43 | GDGEILDEVITRSRGEFRHECSPKITSSTATPTNDPQSLRSPR | 2.1e-013 | 3 |
| ***NIP*** | 1 | 21 | PISGASMNPARSLGPAIVSNQ | 9.2e-375 | 44 |
|  | 2 | 100 | WSYSFIRETDKPVHLMSPPSLSFKHRQTKSNDSQVPDNDLPVGELAGVAVGSAVCITSILAGSRFMQLKQEQLLQHSHCVYYYTQLDVLEPLHLLEQTSK | 2.6e-319 | 5 |
|  | 3 | 100 | PIAKLWQRQPTCWSSHVALQLVQAMSTKLPNEPQLLEDLLSSMRLVTSLVHTTLLSLHLQLDVFPGNRWQQMFTKISIAKLRDKMILSQKAHYHPTGSHC | 8.60E-221 | 4 |
|  | 4 | 15 | WTNHWVYWVGPFIGA | 1.50E-171 | 29 |
|  | 5 | 21 | MEIIITFALVYTVYATAADPK | 1.20E-136 | 33 |
|  | 6 | 21 | LGTIAPIAIGFIVGANILAAG | 1.20E-93 | 25 |
|  | 7 | 21 | RALLYMIAQCLGAICGCGLVK | 3.80E-62 | 15 |
|  | 8 | 20 | GAWVYNMVRYTDKPLREITK | 4.60E-26 | 4 |
|  | 9 | 26 | GAWVYNMVRYTDKPLREITK | 6.40E-23 | 4 |
|  | 10 | 27 | KDYQDPPPASLIDAEELTKWSFYRAII | 5.80E-23 | 4 |
