# Supplementary material for: Boron homeostasis affects Longan yield: a study of NIP and BOR boron transporter of two cultivars
Source: BMC Plant Biol. 2024 Jan 2;24:9. doi: 10.1186/s12870-023-04689-8 (PMC10759464; doi:10.1186/s12870-023-04689-8)
Supplement: Supplementary file 7 — Additional file 7: Fig. 4. Gel electrophoresis of PCR products of transferred genes from transgenic pollens. Transfection of NIP19-GFP and NIP1-GFP pollens was confirmed by amplifying three biological repeats from each and resolving them on agarose gel electrophoresis. [file 12870_2023_4689_MOESM7_ESM.docx]

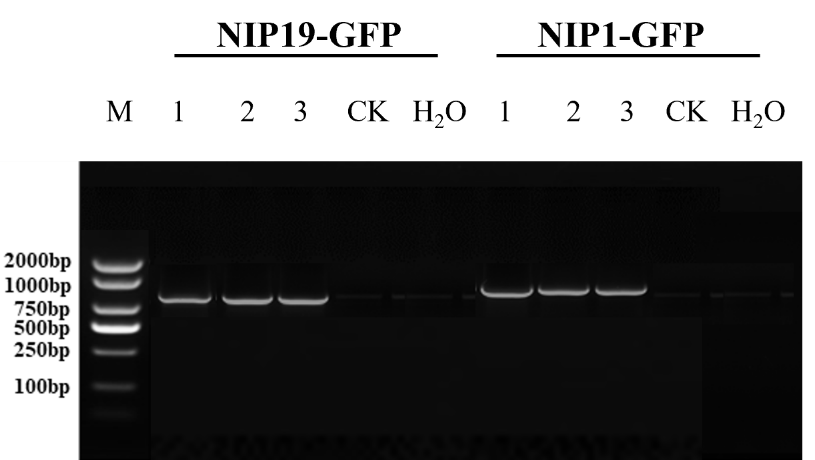


**Fig. 4**: **Gel electrophoresis of PCR products of transferred genes from transgenic pollens.** Transfection of NIP19-GFP and NIP1-GFP pollens was confirmed by amplifying three biological repeats from each and resolving them on agarose gel electrophoresis.
